# Association between change in body weight during early lactation and milk production in automatic milking system herds

**DOI:** 10.3168/jdsc.2022-0323

**Published:** 2023-04-28

**Authors:** Mateus Peiter, Luciano Caixeta, Marcia I. Endres

**Affiliations:** a Department of Animal Science, University of Minnesota, St. Paul 55108; b Department of Veterinary Population Medicine, University of Minnesota, St. Paul 55108

## Abstract

The objective of this observational study was to investigate the association between percent body weight (BW) change in early lactation and the 90-d cumulative milk yield of dairy cows in automatic milking system (AMS) herds. Retrospective daily cow data were collected from the Lely T4C (Lely Industries, Maassluis, the Netherlands) software on 34 farms. Cows were categorized by parity into parity 1 (P1), parity 2 (P2), or parity 3 and greater (P3+). The BW change over the first 21 d of lactation was calculated as the percentage difference between the cow's average BW across d 20 through 22 and the average BW across d 2 through 4 (initial BW) postpartum. The 90-d cumulative milk yield was the outcome variable in a mixed linear regression model, with BW change, parity, their interaction, and season of calving as explanatory variables. Farm and cow nested within farm (n = 4,695) were random effects in the model. On average, cows in all 3 parity groups lost BW during the first 21 d in milk. The 21-d BW change had a negative quadratic relationship with 90-d cumulative milk yield for all parity groups; P1, P2, and P3+ cows with a 21-d BW change of −7.42%, −5.02%, and −4.52%, respectively, were more productive over 90 d in milk (P1 = 3,123 ± 52.6 kg, P2 = 4,271 ± 52.8 kg, and P3+ = 4,548 ± 52.2 kg). The findings of this study highlight the benefits of monitoring BW change in early lactation and may contribute to future research aimed to develop or improve predictive models for milk production in herds using AMS.

## Highlights

• Cows lose weight during the postpartum period as they mobilize body reserves for meeting nutrient demands of milk production.
• Multiparous cows that lost approximately 5% and primiparous cows that lost 7.5% of their initial calving BW during the first 21 DIM were more productive than cows that lost or gained more weight.
• These findings demonstrate the usefulness of data from automated technologies to improve management of transition dairy cows.

## Graphical Abstract

**Figure:**
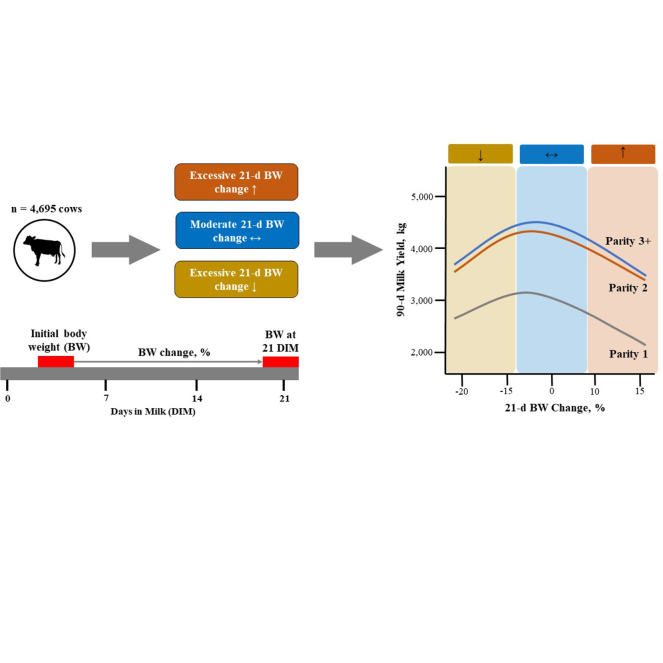
**Summary:** Dairy cows mobilize adipose and muscle tissues as a response mechanism to the increased unmet nutrient demands in early lactation. We wanted to know whether this change in body weight (BW) was associated with milk production. The percent BW change from initial average BW of d 2 to 4 after calving (referred to in the graph as initial BW) to the average of 20 to 22 DIM (referred to in the graph as 21-d BW) was calculated for each cow (n = 4,695). We found that cows that lost approximately 5% (multiparous) or 7.5% (primiparous) of BW over the first ∼21 DIM were more productive than cows that gained weight or lost more weight.

The early lactation represents a challenging period for dairy cows as the rapid increase of nutrient requirements for milk production is rarely met by the DMI in the early postpartum period, resulting in a negative nutrient balance (Drackley, 1999; de Vries and Veerkamp, 2000; Weber et al., 2013). As a response mechanism to this increased unmet nutrient demand, adipose and muscle tissues are mobilized, leading to losses in body condition and weight.

The health and metabolic status of the cow during the first 30 DIM has been shown to affect the BW change differently in different parity groups (Caixeta et al., 2015). A positive correlation was found between liver fat concentrations in the postpartum period and BW loss, and the milk production of cows with different levels of liver fat content was found to be similar (Weber et al., 2013). However, Zachut and Moallem (2017) showed that cows with greater than average BW loss between the first and fifth weeks of lactation had greater milk production during the first 30 DIM compared with cows with reduced BW loss.

Technologies such as the automatic milking system (**AMS**) have the capability of recording individual cows' milk production variables and BW daily rather than less frequently as has been done in most studies in the past. These data from AMS farms could facilitate the identification of animals at greater risk for metabolic disorders and lower milk production and help with management decisions on these farms. Therefore, the objective of this observational study was to investigate the association between percent BW change in the postpartum transition period and 90-d cumulative milk yield. The findings of this study may contribute to future research aimed to develop or improve predictive models for milk production in herds using AMS.

Data were collected from 34 AMS farms in the United States (located in Minnesota and Wisconsin). All farms in this observational study used a free-flow cow traffic system, such that cows had unrestricted access to all areas of the pen, including the AMS. Retrospective daily data were collected from the AMS software (T4C, Lely Industries, Maassluis, the Netherlands) for a period of 12 mo during the years of 2017 and 2018. The data included date, cow identification and respective parity, milk yield, and average BW. The data were recorded during each milking and transformed into daily values per cow by the AMS software. Herds enrolled in this study consisted of Holsteins and all the cows were housed in freestall barns with no access to pasture.

All data management procedures and statistical analyses were performed in RStudio (R Core Team, 2020). Cows were categorized according to parity into parity 1 (**P1**), parity 2 (**P2**), or parity ≥3 (**P3+**). The initial BW was calculated as the average BW across d 2 through 4 after calving and it is referred to throughout the article as initial BW. Day of calving (i.e., d 0) and d 1 postcalving were removed from the data set to minimize the potential effects of retained fetal membranes and lower feed intake around the time of parturition. Similarly, the BW at d 21 was calculated as the average BW across d 20 through 22, and it is referred to throughout the article as 21-d BW. A 3-d average was used for both BW measures in an effort to reduce potential effects of rumen and udder fill. The BW change was calculated as the percentage difference between the cow's 21-d BW and the initial BW. Total milk production per cow was calculated over the first 90 DIM. Season of calving was categorized based on month of calving, where winter consisted of the full months of December, January, and February; spring was considered from March to May; summer included the months of June, July, and August; and the months of September through November were assigned to fall. Milk production varies among seasons; therefore, it was important to account for season in the model. The final data set had variables for farm, cow ID, parity, 21-d BW change, 90-d cumulative milk yield, and season of calving from 4,695 cows with 1 observation each. Number of cows per farm ranged from 20 to 434.

A mixed linear regression model was created for the outcome variable 90-d cumulative milk yield. Due to prior knowledge of a possible quadratic relationship between BW change and milk production, fixed effects included both the linear and quadratic term of 21-d BW change (continuous), parity (3 categories), the BW change × parity interaction, and season of calving (4 categories), along with the random effects of farm and cow nested within farm. The quadratic term for 21-d BW change fitted the data better, so only the interaction between the quadratic term and parity was included. Model fit was assessed by visual observation of residual plots and root mean square error. The denominator degrees of freedom were estimated using Satterthwaite's method. Graphical visualizations of data were created using tools of the ggplot2 package (Wickham, 2016). Significance was declared at *P* ≤ 0.05. The Tukey *P*-value adjustment was used for pairwise comparisons.

We analyzed data from 4,695 cows (P1 = 1,645; P2 = 1,349; and P3+ = 1,701). Initial BW was (mean ± SD) 608 ± 64, 686 ± 69, and 755 ± 76 kg, whereas average BW at d 21 was 581 ± 59, 665 ± 65, and 725 ± 73 for P1, P2, and P3+ cows, respectively. On average, cows in all parity groups lost BW over the first 21 DIM. The least squares means for 90-d cumulative milk yield were highest for P3+ cows, with a production of 4,545 ± 52 kg (LSM ± SE), followed by P2 cows producing on average 4,266 ± 53 kg, and P1 cows with a 90-d milk yield of 3,094 ± 53 kg.

The overall 21-d BW change was −3.65 ± 4.22% (mean ± SD), and −4.25 ± 4.41%, −2.94 ± 4.16%, and −3.64 ± 3.99% for P1, P2, and P3+, respectively. A negative quadratic association between 21-d BW change and 90-d milk yield was found for all parity groups (*P* < 0.0001), and associations differed between parity groups (*P* = 0.01; Figure 1). The association was different between P1 and P3+ (*P* = 0.004), whereas the curve for P2 was not different from P3+ (*P* = 0.57) or P1 (*P* = 0.056). Marginal means for 90-d milk yield were estimated for every decimal point in BW percent change. The curve turning point (i.e., maximum 90-d milk yield) decreased with parity number. The P1 cows who lost an average of −7.42% of their initial BW by ~21 d produced the most milk with an estimated 3,123 ± 52.6 kg (LSM ± SE) over the first 90 d in milk. This would equate to P1 cows with the average initial weight of 608 kg having a 21-d BW loss of 45 kg or 2.5 kg/d. The P2 cows achieved the greatest average production at 4,271 ± 52.8 kg when 21-d BW percent change averaged at −5.02%. This would equate to P2 cows with the average initial weight of 686 kg having a 21-d BW loss of 34 kg or 1.9 kg/d. Last, the greatest 90-d cumulative milk yield for cows in P3+ (4,548 ± 52.2 kg) was achieved when 21-d BW change averaged at −4.52% of their initial BW. This would equate to P3 cows with the average initial weight of 755 kg having a 21-d BW loss of 34 kg or 1.9 kg/d.

**Figure 1:**
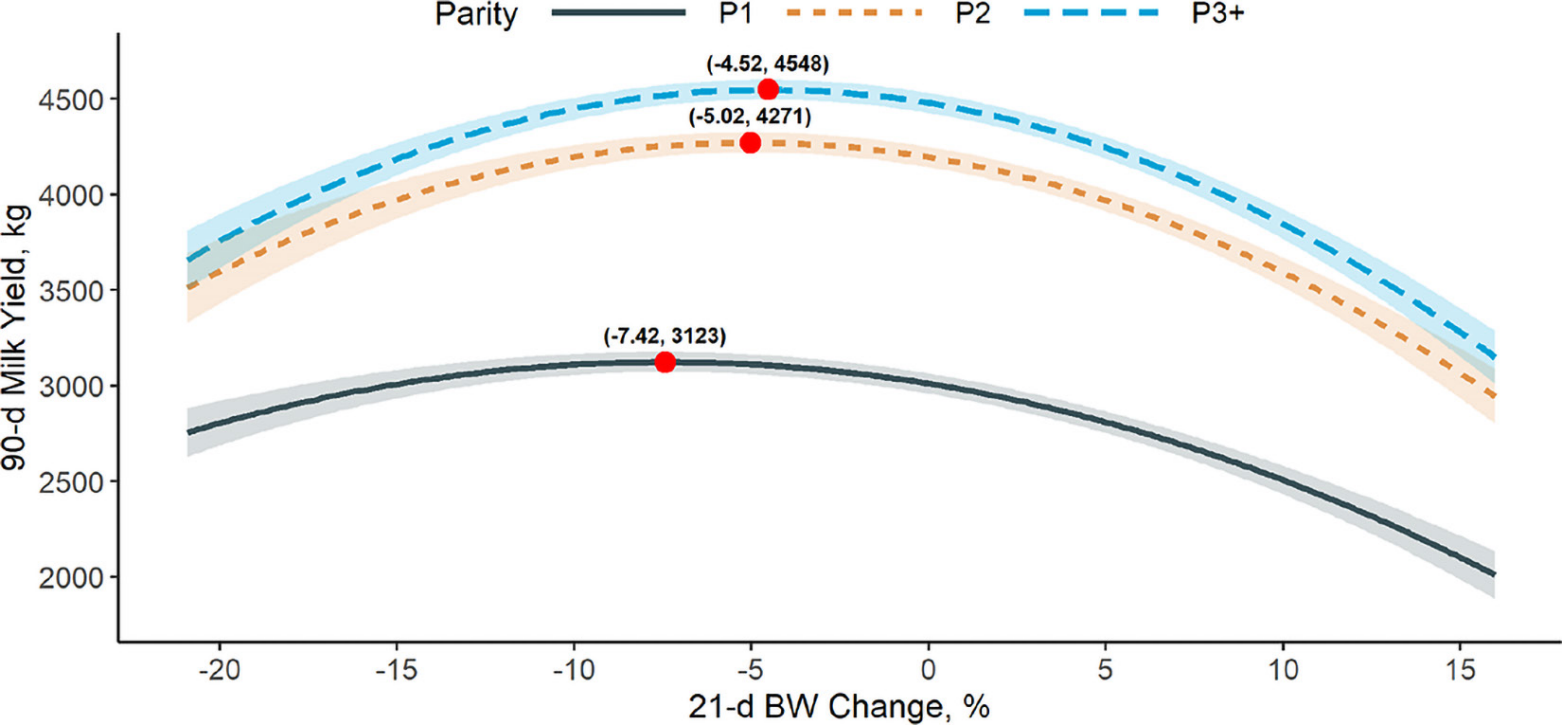
Least squares means and standard errors (transparent bands) of 90-d milk yield (kg) for the interaction between parity and 21-d BW change [% difference between average BW across d 20 through 22 and the average BW across d 2 through 4 (initial BW) postpartum]. Fixed effects model: 21-d BW change + 21-d BW change^2^ + parity + 21-d BW change^2^ × parity + season of calving. *P*-values of fixed effects: 21-d BW change < 0.0001; 21-d BW change^2^ < 0.0001; parity < 0.0001; 21-d BW change^2^ × parity = 0.01; season of calving < 0.0001. Model marginal R^2^ = 0.48; conditional R^2^ = 0.99; root mean square error = 16.8. Parity 1 (P1) = solid black line, parity 2 (P2) = short-dashed orange line, and parity ≥3 (P3+) = long-dashed blue line. Red dots (x, y) represent the BW change % (x) for the maximum predicted outcome value (y). n = 4,695 cows from 34 automatic milking system dairy farms.

The objective of this exploratory study was to investigate the association between BW change in early lactation and the 90-d cumulative milk yield on commercial AMS dairy farms. One of the advantages of automated technologies such as the AMS is the valuable monitoring of the herd through data recorded daily (e.g., milk production and BW) at the individual cow level. In this study, BW measurements were recorded by AMS units equipped with scales every time cows entered the box to be milked, as opposed to single daily or weekly measurements, creating a daily average based on multiple observations, decreasing the effects of rumen and udder fill. In addition, initial and d 21 BW in this study were averaged across 3 d to minimize these effects even further. Frequent on-farm BW measuring showed potential to be used as an approximation metric for nutrient balance of cows in early lactation (Thorup et al., 2012). Heuer et al. (2001) calculated the BW change as the difference between weekly average BW per cow adjusted for rumen fill, which was strongly associated with both calculated and predicted energy balance over 7 independent feeding trials. In fact, adjusted BW change was referred to as a proxy for true energy balance (Heuer et al., 2001).

Our findings showed that most cows in all parity groups lost weight in the early lactation period, in agreement with a previous study (Mäntysaari and Mäntysaari, 2015). Our results also are consistent with Caixeta et al. (2015), who found that all cows in the 3 parity groups lost weight over the first 30 DIM. When Caixeta et al. (2015) measured BW change as a linear regression slope coefficient over the first 30 DIM, animals in P3+ had the greatest BW loss expressed in kg/d. It would be expected that cows in P3+ have a greater BW loss in kg/d as these are normally heavier animals. Therefore, the measurement of BW change as a percentage of the BW of each cow was chosen as a more preferable variable to evaluate the relationship between BW loss and milk production for the current study, as it takes into account the differences in animal frame and stature. The use of BW change as a percentage was also chosen by Zachut and Moallem (2017) as an appropriate representation of early lactation change in BW.

Total 90-d milk yield was chosen as a productivity measurement for being post lactation peak for most cows on AMS farms (Peiter et al., 2021), and therefore capturing the period when cows are most productive. In addition, 90-d cumulative milk yield had a Pearson correlation coefficient of 0.95, 0.99, and 0.99 (*P* < 0.0001) with total milk yield over the first 30, 60, and 120 DIM, respectively.

Based on our findings, cows that maintained their BW or lost up to approximately 10% of their initial BW over the postpartum transition period (i.e., 21 d) were more productive during early lactation (Figure 1). We observed that multiparous cows with extreme BW loss or BW gain (i.e., BW change greater than approximately ±10% over 21 DIM) experienced a significant impairment in their 90-d milk yield. For instance, P3+ cows that lost approximately 21.4% of their initial BW over 21 DIM were estimated (LSM ± SE) to have a 90-d milk yield of 3,603 ± 162.0 kg, and cows in the same parity group with a BW gain of 16.7% had an estimated 90-d milk yield of 3,037 ± 149.8 kg. If we compare those numbers to the 4,548 ± 52.2 kg of P3+ cows with a 21-d BW loss of 4.5%, the severe impairment in milk production of cows on both ends of the curve is clear. The maximum estimated 90-d milk yield for P1 cows was reached when they lost 7.4% of their initial BW, and greater BW loss showed not to have a major impact on 90-d milk yield. Primiparous cows that gained BW over the 21 DIM, on the other hand, had a considerable drop in 90-d milk yield. For instance, P1 cows with a 16.8% BW gain over 21 DIM had an estimated 90-d milk yield of 1,932 ± 134.4 kg, as opposed to 3,123 ± 52.6 kg for the ones with an 7.4% BW loss during the same period. The influence of partitioning of nutrients for growth rather than milk production in P1 cows could contribute to this finding and warrants further investigation.

Berry et al. (2007) reported that cows that lost 100 kg from calving to BW nadir produced an average of 139 kg more milk during the first 60 DIM compared with cows that lost 50 kg during the same period. Similarly, ECM yield was greater for cows with greater BW loss and nonesterified fatty acid concentrations in early lactation (Carvalho et al., 2014), suggesting that high-producing cows mobilize greater body reserves to meet the demands of producing more milk. Moreover, greater BW loss in early lactation was associated with increased lactation persistency and higher and earlier peak milk yield (Berry et al., 2007). Therefore, to some extent, our results agree with previous studies, where cows with BW loss in early lactation are those producing more milk. However, we suggest that multiparous cows with extreme BW loss in the postpartum transition period may be the ones that experience a more severe negative nutrient balance and potentially health issues that can also affect DMI, which end up having a negative effect on their productivity.

Zachut and Moallem (2017) showed that 30-d milk yield was 1.4 kg/d greater for cows with high weight loss (7–17%) compared with cows with low weight loss (3–6%) across parities during the first 5 wk of lactation. This finding conflicts with the results of the current study, where we found that especially multiparous cows with >5% BW loss during the first 3 wk in lactation had an impaired 90-d milk yield. However, differences in data management and modeling could explain these differences. In the current study, BW change was a numeric continuous variable, whereas Zachut and Moallem (2017) created 2 BW loss categories. The approach used in our study allowed us to model the data differently and explore a quadratic relationship between BW change and milk yield, highlighting differences hidden by data dichotomization.

The conditional coefficient of determination (R^2^) for the model evaluating the association of 21-d BW change and 90-d cumulative milk yield was 0.99, which means the model explained most of the variability in 90-d milk yield. The fixed effects of BW change, parity, and season explained 48% (marginal R^2^) of the total variability in 90-d milk yield. The root mean square error for the model was 16.8.

Findings of the current study highlight the importance of closely monitoring BW of dairy cows during the challenging early lactation period. In addition, the results may inspire future research aimed at the development of predictive models of milk production based on early lactation BW changes. It is important to consider the effect that rumen fill might have on some of the estimates of BW as cows will be increasing their intake in early lactation, which is a limitation of this field study. In future studies, the influence of individual cow factors such as rumen fill could be considered in the analysis.

Our results indicate that BW change over the first 21 DIM had strong quadratic associations with total milk production over 90 DIM, with differences between parity groups. Findings suggest that highest-producing primiparous cows lose 7.4% of their BW over the first 21 DIM, whereas those with extreme BW gain had a much lower milk production over 90 DIM. Additionally, the highest-producing multiparous cows on AMS farms are the ones losing an average of approximately 5% over 21 d. These findings demonstrate the usefulness of data from automated technologies to improve management of transition dairy cows.
